# Micro-credentials in leveraging emergency remote teaching: the relationship between novice users’ insights and identity in Malaysia

**DOI:** 10.1186/s41239-022-00323-z

**Published:** 2022-04-01

**Authors:** Jeya Amantha Kumar, Rachel Jasmine Richard, Sharifah Osman, Kevin Lowrence

**Affiliations:** 1 Centre for Instructional Technology and Multimedia, Universiti Sains Malaysia, Pulau Pinang, Malaysia; 2 Keysight Technologies, Bayan Lepas, Pulau Pinang, Malaysia; 3 School of Education, Faculty of Social Sciences and Humanities, Universiti Teknologi Malaysia, Johor, Malaysia; 4 Faculty of Education, Universiti Kebangsaan Malaysia, Selangor, Malaysia; 5 International Admissions, Northwood University, Midland, MI USA

**Keywords:** Micro-credential, Digital badges, Digital learning identity, COLLES, Higher educational institutions, OpenLearning, Pre-service teachers, Malaysia

## Abstract

Micro-credentials have gained much popularity in recent years, and their popularity has skyrocketed due to emergency remote teaching instigated by the pandemic. It has been defined as a platform that provides credentials based on validated competencies. Nevertheless, in Malaysian HEI, such a concept is still novel and identifying insights on the benefits, challenges, and application are still scarce. Similarly, it was observed that there is a lack of observation on how students’ digital learning identity and their perception of professional relevance are influenced by such platform. Henceforth, based on the adapted enriched virtual model approach, a micro-credentials course was implemented to complement the new “normal” classes for a pre-service teacher’s instructional design course. A mixed-method triangulation design was used to explore the qualitative findings operationalized by open-ended questions (N = 74) with data obtained from the Digital Learning Identity Survey (DLIS) and Constructivist On-Line Learning Environment Survey (COLLES) (N = 72). The findings indicated that respondents had an overall positive perception of the use of micro-credentials to complement and overcome online learning challenges mainly due to substandard internet connectivity; nevertheless, they are unaware of the value of such credentials in their future profession. Conversely, their new identity as digital learners and experiences with a blended approach of online learning, especially with micro-credentials, was successful in shaping their identity as aspiring educators that embrace technology for teaching and learning.

## Introduction

In view of the current COVID-19 pandemic, education institutions are on the cusp of fully online learning to deliver lessons and training as traditional teaching and learning methods are faced by challenges (Tang et al., 2021). It cannot be denied that the continuum of education is vital despite the pandemic and virtual or online learning seems to be the only alternative. Nevertheless, it is also undeniable that before the shift, there has been an exponential rise in open education resources (Clements et al., 2020) such as massive open online courses (MOOCs) namely Coursera, EdX, and FutureLearn (Chakroun & Keevy, 2018; Kumar & Al-Samarraie, 2019) where such platforms also offer credentialing through structured microlearning strategies (Peacock et al., 2020) that are interactive, self-directed, and flexible (Lim & Hassan, 2020). Completing learning activities through these platforms are further recognized through various types of credentials such as certificates, digital and open badges (Selvaratnam & Sankey, 2021) that represent a competency or skill attained usually based on a network of evidence-rich credentials (Willis, Strunk, et al., Willis, Strunk, et al., 2016).

As a result, the MOOC platforms have inadvertently orchestrated the rise of micro-credentials (Wheelahan & Moodie, 2021) especially through industry and third party with collabration. Initially, it was strategized to accommodate the rising cost of formalized learning (LaMagna, 2017), however due to employers’ cynical questioning on the validity of traditional competencies (Gauthier, 2020), micro-credentials stepped up as an alternative path for lifelong learning. Henceforth, as a disruptive innovation (Lim et al., 2018) even before the pandemic, it has been trusted as a recovery strategy to overcome learning mobility issues especially during Covid-19 (Wheelahan & Moodie, 2021). Therefore, leveling it to cater for upskilling (Eager & Cook, 2020) and reskilling (Cirlan & Loukkola, 2020) which are essential for the ‘new normal’ (Boud & Jorre de St Jorre, 2021). As an example, Coursera’s version of micro-credential is known as MasterTrack Certificate and Professional Certificate; edX offers XSeries, MicroMasters, Professional Certificate and Professional Education; Udacity offers Nanodegree.

In hindsight, it is unsurprising that micro-credentials may potentially expand post-COVID due to the need to rapidly adapt, equip and credit students with beneficial non-degree skills before entering the workforce (Penn State Online Coordinating Council, 2020). According to Ruddy and Ponte (2019), higher educational institutions (HEI) students are now considering credentials that increase their chances of employment that are easily acquired while adding value to their academic credentials. Nevertheless, as promising as micro-credentials are as a disruptive technology for teaching and learning, there has been limited studies on its pedagogical implementation, adoption (Clements et al., 2020), and strategies of sustaining it, especially for HEI (Selvaratnam & Sankey, 2021). Furthermore, the use and value of micro-credentials to provide competencies (Hunt et al., 2020; Young et al., 2019), its role in higher education and employability is still under research (Pickard et al., 2018). Ideally, these credentials should be transferable through professional platforms and university transcripts (Lim et al., 2018; Ruddy & Ponte, 2019). Nevertheless, we are still far from this expectation. Furthermore, some of these organizations do not require HEIs for skills validation as big-tech giants such as IBM, Microsoft, and Oracle provide their own professional credentials such as Google’s Data Analytics Professional Certificate or Microsoft Certified Professional. Hence, placing HEI in a *rat race* to either provide and recognize their own credentials or partner with industries that can fulfill these needs for workplace recognition.

In Malaysia, the micro-credential movement in HEIs is relatively new, with accreditation strategies by the Malaysian Qualifications Agency (MQA) being formally implemented only in 2019 (Malaysian Qualifications Agency, 2019). Henceforth, there have been limited studies on its implementation due to its untested approach. Nevertheless, according to West et al. (2020), such limitation provides an opportunity for researchers to explore the role of micro-credentials in a wider context. Besides, empirical findings by Lim et al. (2018) highlighted the need to investigate how micro-credentials are accepted and understood when introduced in Malaysian higher education. On the other hand, at present, both educators and learners are faced with challenges of using online learning platforms due to the “new normal”; hence, we defined this as a new identity as a learner. Zimmer et al. (2021) defined it as digital learning identity (DLI) developed through self-perception of personal skills and beliefs used to overcome challenges in digital learning. We theorized that understanding their identity as a learner and the usefulness and relevance of the course towards their professional growth is fundamental in investigating the effectiveness of the intervention. This is further supported by Willis, Strunk et al. (Willis, Strunk, et al., 2016) emphasizing investigations on the relationship between identity and enrollment in micro-credentials. Conversely, to date, we have not found any study that highlights learner identity namely a digital learner nor professional learner in influencing their perception of partaking in micro-credential.

Furthermore, as micro-credentials can be delivered either online or through a blended approach (Cirlan & Loukkola, 2020), we aim to investigate its role in facilitating learning as the traditional classroom (brick-and-mortar campus) has been replaced by virtual classrooms conducted through video conferencing tools. This shift is known as emergency remote teaching (ERT) and Bond et al (2021) described ERT is an unplanned strategy of teaching and learning where any available online and offline resources are used to facilitate teaching during the onset of the pandemic. Recent studies of blended approach such as by Hew et al (2020), have re-strategized the face-to-face practice by transforming it to a fully online approached by using video conferencing and collaborative tools out of necessity. Therefore, we strategized this study to be orchestrated based on blended approach by adapting the enriched virtual model by focusing on exploring the initial perceptions and digital identity of HEI students towards micro-credentials. Henceforth, the primary aim of this study is to investigate how micro-credentials can complement current learning strategies as an alternative platform that supplements courses that have gone fully online while still reflecting its attributes.

## Literature review

### Micro-credentials

Micro-credentials are credentials that acknowledge an individual’s accomplishments for a specific skill (Clements et al., 2020). Traditionally, all credentials obtained from institution-driven educational programs are defined by CGPA or grades indicated through a degree or diploma transcript referred to as macro credentials (Randall & West, 2020). However, micro-credentials do not represent a comprehensive interrelated skill reflected by a degree or diploma program but embody the proficiency of some skills (Ehlers, 2018; Pickard et al., 2018) through a lifelong learning ecosystem (Kift, 2021). It integrates learning needs by balancing work and life challenges to create meaningful learning experience (Ponte & Saray, 2019) that are sustainable, authentic and learner autonomy (Peacock et al., 2020). Lim et al. (2018) defined micro-credentials as an innovative form of short courses used for upskilling to ensure competence for employability.

Accordingly, there are a variety of terms used to encompass the micro-credential spectrum, such as alternative credentials, short courses, digital badges, professional certificates, non-degree certificates (not issued by a university), nano-credentials, MOOCs, and accredited programs (Peacock et al., 2020; Raish & Gross, 2021) and the most common association has been towards digital badges and certification, such as completion and participation. Henceforth, while it has become habitual to refer to micro-credentials as digital badges, badges have the same purpose with certificates where they encompass meta-data such as course details and participant information used to document, communicate (LaMagna, 2017), and share achievement credentials across different platforms (Lim & Hassan, 2020). Fanfarelli and McDaniel (2019) added that besides credentialing, digital badges have been regarded as motivational and can be used to recognize work in progress in a course.

All the same, the application of digital badges is restricted if the badges are not recognized and validated through a professional network that is able to communicate achievement (West et al., 2020). While this predicament is seen as challenging, the unavailability of a mutually recognized global credentialing system created an opportunity for industries, professional bodies, and licensing organizations to offer credentials that are recognized worldwide. (Chakroun & Keevy, 2018). Henceforward, the introduction of open badges standards by the Mozilla Foundation in 2011 as a specific method of packaging pieces of information as an image to represents validated and verified accomplishments. These badges rely on a common standard of open-source technology known as the Open Badge Infrastructure (currently under the stewardship of IMS Global) (West et al., 2020) that allows credentials to be interchanged between different systems (Fanfarelli & McDaniel, 2019) especially between conventional or non-academic environment (Raish & Gross, 2021). However, while micro-credentials may provide opportunities to remove barriers between formal, informal, and professional education (Clements et al., 2020), such terms are often not significant from the employers’ point of view especially if it voids an industry recognized learning path (Randall & West, 2020). Interestingly, Wheelahan and Moodie (2021) projected micro-credential as imperative part of future higher education policy.

### Micro-credentials in HEI

According to Ruddy and Ponte (2019), the initiation of micro-credentials is changing the higher education landscape and HEIs are working towards implementing and acknowledging micro-credentials as a new learning path. In an era where there is a continuous change in knowledge and skills, a degree program—with pre-determined courses—of three or four years may not enable students to grasp the respective requirements for job employability (Selvaratnam & Sankey, 2021). Lim et al. (2018) added that while HEIs are supposed to be responsive and industry-driven by being ensuring new skills and credentials are affordable and conveniently acquired; this goal is often unmet.

Henceforth, there has been movement on how to strategize micro-credentials effectively in HEI by ensuring adaption of contemporary and flexible approaches through reformation of policies and taxonomies. In Europe as an example, a study done by MicroHe (2019) on methodological and operational challenges of micro-credentials reported a need for a Recognition-Framework for Micro-Credentials by exploring the relationship between *student* (holders of micro-credential), the *university* (issuers), *employers* (enquirers) and *regulators* (defined standards). A more recent project named MICROBOL: Micro-credentials commissioned by the Erasmus + Programme of the European Union (Cirlan & Loukkola, 2020) identified micro-credential as having the following characteristics:

i. it can be offered or recognized by higher education institutions based on standard recognition procedures
ii. the skills, knowledge, or competencies should respond to societal, personal, cultural, or labor market needs
iii. explicitly defined learning outcomes in accordance with the national quality framework
iv. recommendation of workload or learning time based on a transferable educational credit system
v. assessment methods and conditions
vi. subject to quality assurance in line with a quality framework

Henceforth, it is evident that micro-credentials contribute to how we define and structure teaching and learning in HEI by emphasizing frameworks and standardization besides merrily content creation (Wheelahan & Moodie, 2021). Nevertheless, this is one of the main issues, as HEI’s are often bounded by educational accreditation requirements set by the governing bodies that on one side provides quality assurance but on the flip side restrict content development and implementation strategies. Stefaniak and Carey (2019) further added that issues in HEI can be often rooted on usability, applicability, and lack of understanding of its goal and value. Therefore, without a framework to guide and validate the value of such strategies, the incompetence of such intervention becomes apparent. On the other hand, micro-credentials is a clear option in helping HEI students validate their abilities that are not necessarily visible through their academic transcript (Randall & West, 2020). While micro-credential can be implemented based on several approaches such as a mixed and matched approach (hybrid) or by complementing conventional courses (Boud and Jorre de St Jorre, 2021), it depends on the creativity and value projected to ensure its success. Besides, while it aids validation of students’ skills that enable them to demonstrate their competency (Lim et al., 2018), credential stackability also allows them bundled or unbundled credentials to achieve a new learning goal (Pickard et al., 2018).

### Micro-credentials in Malaysia

Micro-credentials are relatively new to the Malaysian education system, and in conjunction with implementing micro-credentials, Universiti Sains Malaysia (USM) is pioneering such a strategy in collaboration with OpenLearning.com. Henceforth, USM is in the midst of designing and developing the USM’s Micro-credential Online Learning Portal, which provides support to learners, educators, and organizations or industries geared toward obtaining skills and competencies. It is highly encouraged for micro-credentials to be designed to meet the requirement of the industry by collaboration and courses to complement online learning. Therefore, to support such endeavors the Malaysian Qualifications Agency (MQA) published a guideline on micro-credential implementation, its justification, principles, and best practices in Malaysia (Malaysian Qualifications Agency, 2019) by which it has outlined two main focuses:

i. digital verification (digital badges, digital, nano degrees, micro degrees) that are transparent, secure to avoid misuse, and shareable via social media platforms, email, blogs, and resumes
ii. records the achievement of learning of a specified set of outcomes (knowledge, skills, attitudes) which are outcome-based, personalized and industry-driven

The guideline justifies the need for micro-credentials to promote easy access to life-long learning through dynamic, demand-driven, stackable, shorter, and cost-efficient credentialing that recognizes the need for non-formal learning. Henceforth, the guideline also emphasizes an effective Quality Management System (QMS) to be placed to ensure strategic management, monitoring, and validation of micro-credentials. While the movement to establish a national framework is still new, Taylor’s University proposed a university-level framework in 2018 (Lim et al., 2018), focusing on transforming teaching and learning strategies for their academic professional development (Lim & Hassan, 2020). Nevertheless, to date, most institutions are promoting internal courses or partnering with international and local governing bodies. An example is Tunku Abdul Rahman University College which is a private HEI in Malaysia is offering micro-credentialling courses recognized by the Malaysia Digital Economy Corporation (MDEC) for Artificial Intelligence, Database Management (Oracle Academy Database Design and Programming with SQL curriculum), and Introduction to Information Technology (CompTIA A + professional certification and Cisco Networking Academy IT Essentials curriculum). Besides that, Asia E University as an Authorised Training Partners (ATP) has also partnered with iTrain Asia, which is a certification body covering 16 countries in Asia, The National Tech Association of Malaysia (PIKOM), and the Human Resource Development Fund (HRDF). The collaboration is to design Malaysia’s first Digital Tech Micro-Credentials Programme that focuses on reskilling non-tech individuals in Data Engineering, Full-stack Development, UX Design, and Deep Learning (AI) through Micro degrees.

Nevertheless, industry recognized credentialing often does not fulfill the Accreditation of Prior Experiential Learning (APEL) and credit transfer requirements for formal education in Malaysia. Hence, we also observe that there are two paths of credentialling that are independent of each other, namely, by either providing formal education or industry upskilling opportunities and there is a partial link that associates both paths. However, in Malaysia, the OpenLearning platform has provided an alternate solution by implementing the OpenCreds Framework in Malaysia (15th October 2020), which mirrors the credentialing (OpenCreds Australia) of Open Universities of Australia as an initial step towards credit transfers across different countries and industries. To date, the OpenCreds is in accordance with the Malaysian Qualification Framework (MQF) with flexibility and options for interoperability and “stacking” as it has highlighted a fixed credits awarding system based on learning hours per credit system (Openlearning, 2020).

Conversely, such a strategy while fulfilling the credit requirements of HEI can also be used for accomplishing Continuing Professional Development (CPD) hours. However, as described earlier, the trends of courses being offered are parallel with Shah (2020), claiming the majority of micro-credentials (73%) focusing on business and technology, with only 2.3% focusing on education and teaching. Nevertheless, Hunt et al. (2020) and Lim and Hassan (2020) described micro-credentials as be fundamental in catering to the need and goals for teachers’ professional development. By so, corporations such as Teach for America and Purdue University have used it to validate educators’ competencies (Randall & West, 2020). Furthermore, micro-credentials have great potential to complement conventional courses for lifelong learning (Cirlan & Loukkola, 2020), and we target to explore how such avenues would influence pre-service teachers’ perception.

### Research aim

According to Ralston (2021), while there seem to be different values between micro-credential and university degrees, they can coexist side-by-side. Therefore, due to the novelty of micro-credential studies, it is imperative to investigate the implementation, transferability, and sustainability of micro-credential ecology to ensure meaningful learning (Selvaratnam & Sankey, 2021) by identifying challenges faced by novel users (Ellis et al., 2016). Moreover, exploring micro-credential would also gain by examining relationships between credentialing with developing professional identity (Young et al., 2019) and digital literacy skills (Ruddy & Ponte, 2019); hence we identified two separate identities that should be considered which is the identity as a digital learner and also a professional.

Furthermore, as mentioned, there are limited applications of micro-credentials specifically for the field of education and training (Shah, 2020) and this is more apparent considering the novelty in Malaysia. Nevertheless, initial perception and belief are also fundamental in determining the future success of an online learning tool (Kumar et al., 2020), as novice users usually provide insight on user experience gaps that might be overlooked by the system designer (Walker & Prytherch, 2008). Retrospectively, to our knowledge, no studies have investigated how professional, nor digital learner identity relates to micro-credential which we believe is vital for the adoption and success of micro-credential implementation. Besides, with the onset of the pandemic, innovative approaches should be implemented to foster learning and overcome traditional online challenges; thus, we investigated how a blended approach, namely using the enriched virtual model (Staker & Horn, 2012), which is novel on its own (Vijayakumar et al., 2020) may be adapted during ERT as an implementation strategy to gather insights related to micro-credential acceptance. Therefore, for that reason, this study explores the value of micro-credentials as a learning alternative during the pandemic from pre-service teachers’ perspectives due to the availability of a full course and purposive sampling.

## Methodology

### Learning approach

The implementation strategy for the micro-credential is based on a blended learning approach centered on the enriched virtual model (Staker & Horn, 2012) and was previously known as online driver model (Horn & Staker, 2011). This model represents strategies for full-time online learners to complete most of the coursework online (outside the classroom); however, they still attend face-to-face learning sessions (Moreira & Ferreira, 2021) where the teacher delivers the curicula while providing optional brick-and-mortar campus exepereinces (Staker & Horn, 2012). In this study, the instructor met the students weekly for a face-to-face session using video conferencing tools to deliver the curricula, and the micro-credentials link was deployed systematically based on the weekly teaching and learning contents through the institutes’ Moodle Platform. Completion of the micro-credentials was validated through the submission of the certificate of completion. Therefore, we adapted this model as it well-matched the teaching and learning approach during ERT exclusively for remote learning when the whole program is fully online and face to face has been replaced by ‘virtual face to face’ approach through video conferencing. Furthermore, such a strategy has been encouraged during the pandemic when physical interaction is impossible (Mahmud, 2020; Wang et al., 2020).

### The learning environment

**Fig. 1:**
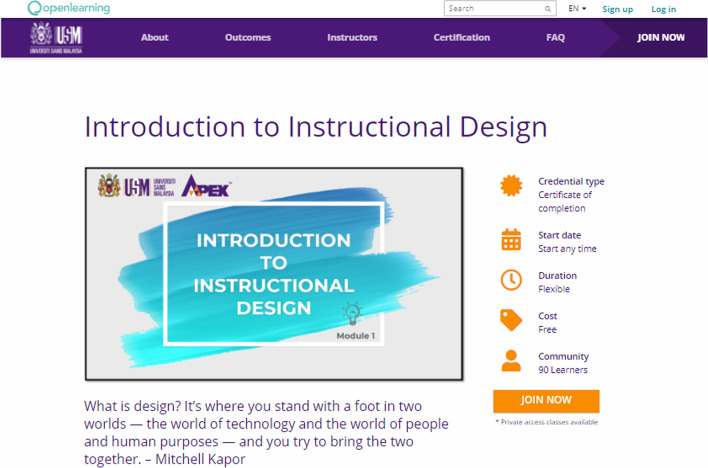
Screenshot of the OpenLearning platform offering the course

The micro-credential course used in this study is named Instructional Design, and it is offered under the micro-credential movement by Universiti Sains Malaysia (USM) for the Bachelor of TESOL or Teaching English to Speakers of Other Languages undergraduates who are also minoring in multimedia. The course aims to provide fundamental skills in designing effective multimedia instructional materials and covers topics such as need analysis, instructional analysis, learner analysis, context analysis, defining goals and objectives, developing instructional strategy and materials, developing assessment methods, and executing them through formative and summative assessments. It is available through the *learning4life* platform hosted by USM using the OpenLearning platform as the foundational learning management system (Fig. 1).

**Fig. 2:**
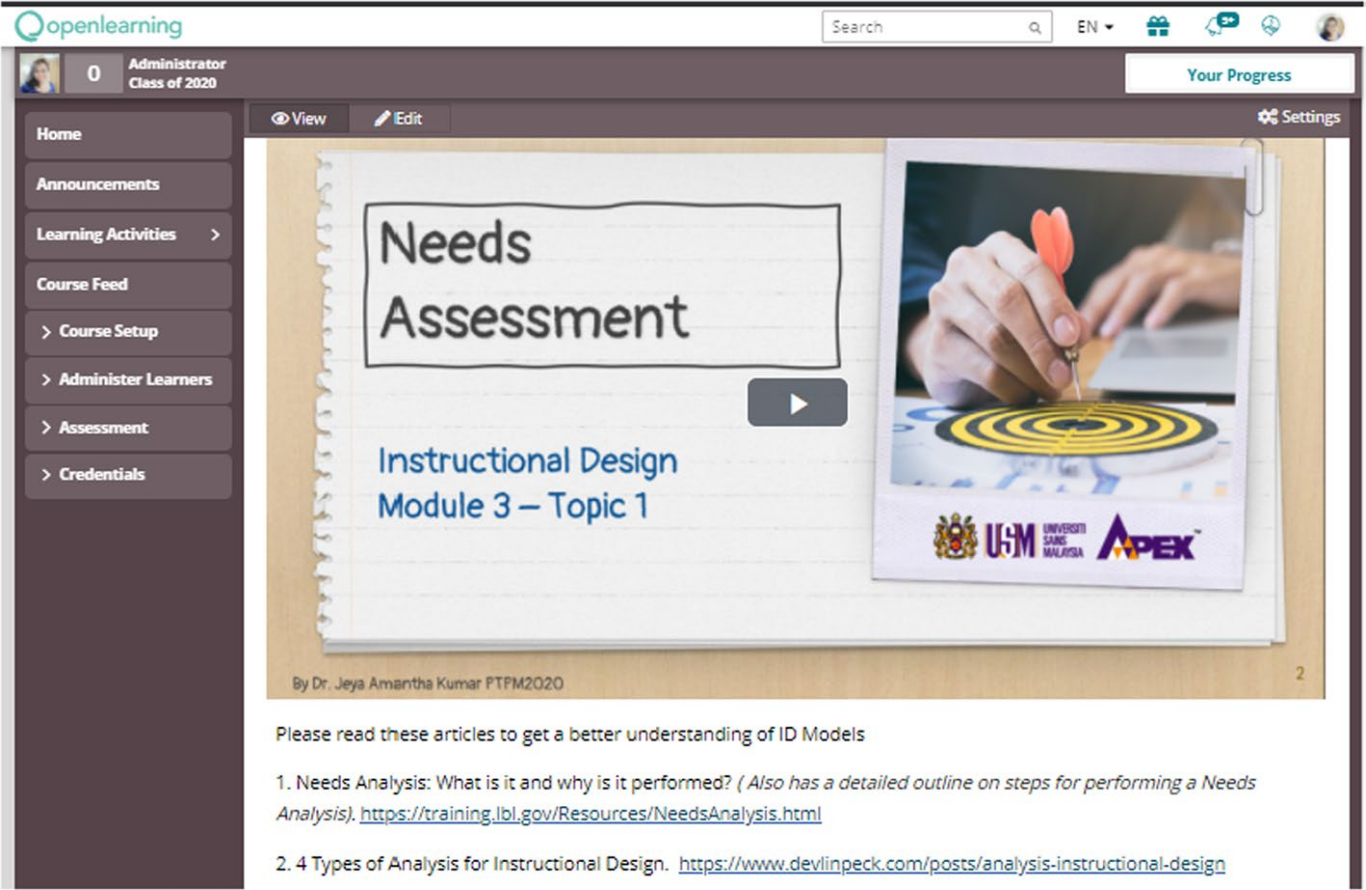
Micro-credential videos and reading material

**Fig. 3:**
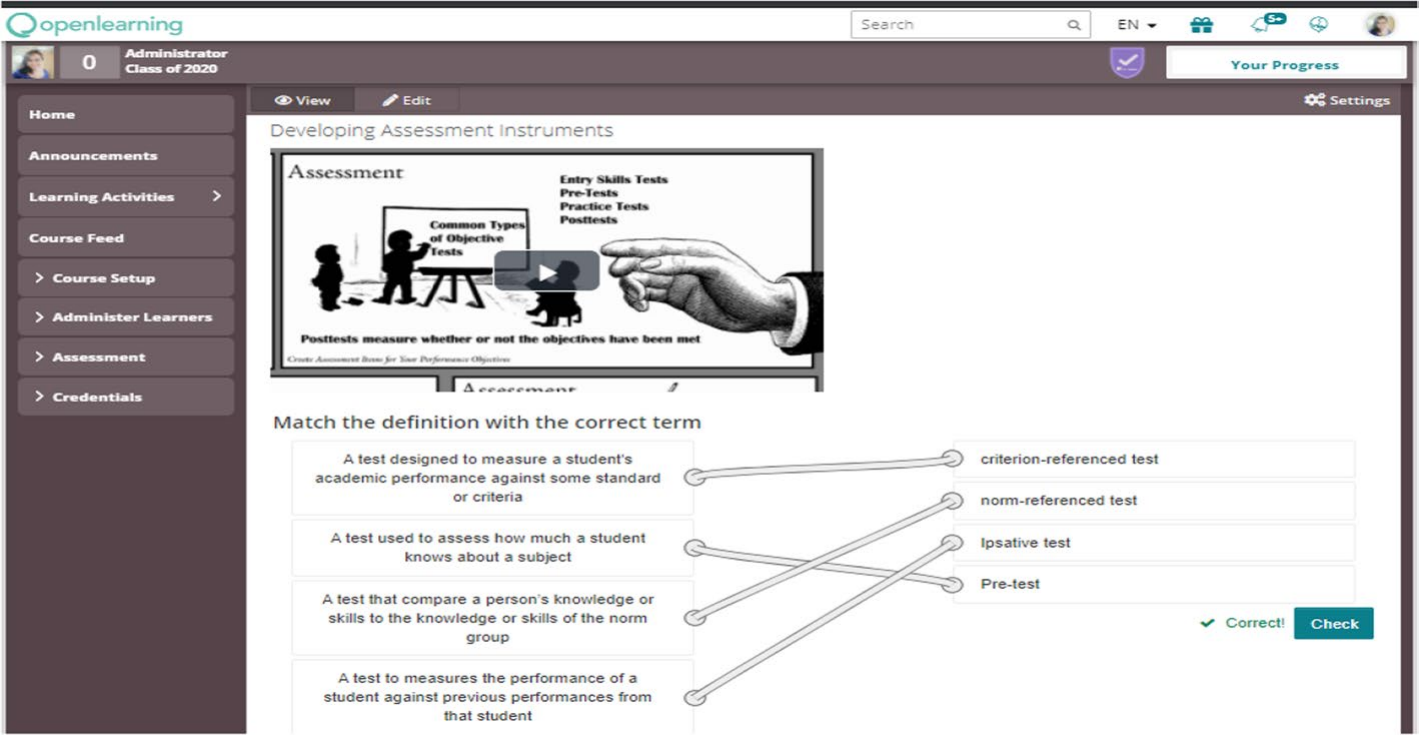
Micro-credential assessment

In total, there are nine modules, namely Introduction to Instructional Design, Instructional Design Models, Needs Assessment, Instructional Analysis, Entry Behavior in Instructional Design, Performance Objectives in Instructional Design, Criterion-referenced Test in Instructional design, Instructional Strategy, and Evaluation in Instructional Design. Each module is freely available, delivered online, certified by the institution, and flexible, provided if the instructor authorizes an access code. Each module is designed based on bite-size learning, where the contents were designed to include lecture videos, creative commons videos (Fig. 2), reading materials, and non-graded assessment (Fig. 3). The completion time of the course was aligned with the planned semester syllabus. Furthermore, without completing one module, the learner cannot progress to the next, as each module is set as a prerequisite to the latter. The completion of each task will be reflected as a digital badge by which a completion certificate will reflect the accumulation of these badges and the module completion.

### Respondent

The total population of the study was 80 students from the Bachelor of TESOL program, and all students were required to participate in the study based on purposive sampling as they were already enrolled in the course. In addition, the single exploratory case study was used to denote the one course purposefully due to the availability of resources for the course. All students were between the ages of 19 and 24 years old, and most of the students in this course are female.

### Research design

Qualitative research was used to justify this exploratory approach to understand the context of the application, addressed issues (Creswell & Poth, 2018), experiences, and perspectives (Hammarberg et al., 2016) through a case study (Creswell, 2015). Furthermore, the exploratory approach was taken due to the study's novelty as there is no clear single set of outcomes regarding the propositions due to lack of knowledge or information of the application (Baxter & Jack, 2015) in the Malaysian context. The data were triangulated with quantitative data to strengthen the reliability and internal validity of the study (Creswell & Creswell, 2018), and this is imperative for investigating novel studies (Fusch et al., 2018) using case study approach (Baxter & Jack, 2015). This mixed-method study is based on a confirmatory design where the quantitative data is used to confirm the qualitative data (Small, 2011). Next, as the qualitative methods are dependent on the subjective perceived view, the feedback will be analyzed using the text analysis approach to create structured data out of the free text content. Text analysis is the process of identifying information using text-mining by revealing patterns and relationships (Schubert et al., 2018). In this study, the analysis is done through computerized text analysis software (https://voyant-tools.org/) to represent trends and themes. Whereas the quantitative data were analyzed using IBM SPSS Statistics 26.

### Instruments

This study used open-ended questions to determine the student’s perception of using micro-credentials to complement their course. In tandem with that, three questions were asked:

RQ1: In your opinion, how do you perceive you have benefited from using micro-credential as a learning platform to complement this course?

RQ2: In your opinion, what were the challenges you faced?

RQ3: In your opinion, do you think the contents you have learned would be valuable to you in your future profession?

We explored two quantitative aspects: digital learning identity (DLI) and professional relevance of online learning to validate the qualitative outcome. Firstly, the DLI survey (DLIS) developed by Zimmer et al. (2021) was specifically designed for the use of the teaching profession to explore how they view digital learning. DLIS identified respondents (1) *attitude* on the importance of technology for learning (2) *self-regulated learning* as perceived ability in monitoring learning with technology (3) *efficacy* as their self-perception on technological proficiency (4) *knowledge achievement,* which reflects using technology for higher purposes of learning (5) *challenge* as difficulties in using technology for learning and (6) *knowledge source* where technology is reflected as a source that signifies knowledge attainment. A five-point Likert scale ranging from “strongly disagree” to “strongly agree” for this 60-item questionnaire was used to measure the DLI.

Next, to measure students’ perception of the micro-credential based on how it is useful and relevant, the Constructivist On-Line Learning Environment Survey (COLLES) (Taylor & Maor, 2000) adopted. Six factors were evaluated (1) *professional relevance* as in how online engagement is relevant to their professional practice, (2) *reflective thinking* on the usefulness of the system, (3) *interactivity* as to the extent to which students engage in educative dialogue (4) *tutor support* which reflects instructors role in enabling online participation (5) *peer support* and (6) *interpretation* which is the extent to which students and instructor make sense of their online learning connection (Mousavi et al., 2020). A five-point Likert scale was used, ranging from “strongly disagree” to “strongly agree” to evaluate the 24 items in COLLES.

### Study procedure

The open-ended questions were embedded in the last modules and the response was voluntary where it was not made compulsory to answer the question. Next, the feedback was extracted from the platform after two weeks to ensure all students were given equal opportunity to provide their feedback. Similarly, COLLES and DLI were executed through Google Forms in consecutive weeks. The extracted data were downloaded as a CSV file and converted to an excel sheet for the analysis process.

The qualitative data from the open-ended questions were analyzed using Voyant Tools (Sinclair & Rockwell, 2021) to establish the emerging themes as a form to analyze content when data sets are large and time-consuming (Hetenyi et al., 2019). Out of the 80 respondents, 74 students provided feedback for the open-ended questions and due to a large number of qualitative responses, text analysis was used. According to Creswell and Creswell (2018), text analysis is part of the qualitative method and can validate the findings from the open-ended questions. In this study, two tools from Voyant Tools were used, namely the Cirrus Tool and the ScatterPlot Tool. The Cirrus Tool is similar to a word cloud representing most frequent terms, whereas the ScatterPlot is a graphical visualization of word clusters analyzed using t-SNE (t-Distributed Stochastic Neighbour Embedding) analysis. The t-SNE is a nonlinear algorithm primarily used in machine learning to process high-dimensional data sets such as qualitative textual data in low-dimensional space (Maaten & Hinton, 2008). In this study, t-SNE is used as the output analysis, whereas frequency setting was based on Term Frequency—Inverse Document Frequency (TF-IDF) which defines the importance of a word to a document. Furthermore, the level of perplexity was maintained as 15, and iteration at 1500 as per the system's settings. Next, the quantitative data for DLIS and COLLES obtained from the Google Form responses were downloaded as an excel spreadsheet and analyzed individually to determine reliability, mean value, and correlation. The quantitive findings will be used to validate the qualitative findings obtained from the content analysis.

### Findings

The findings of this study will be discussed based on the qualitative approach followed by the quantitative approach.

### Content analysis

**Fig. 4:**
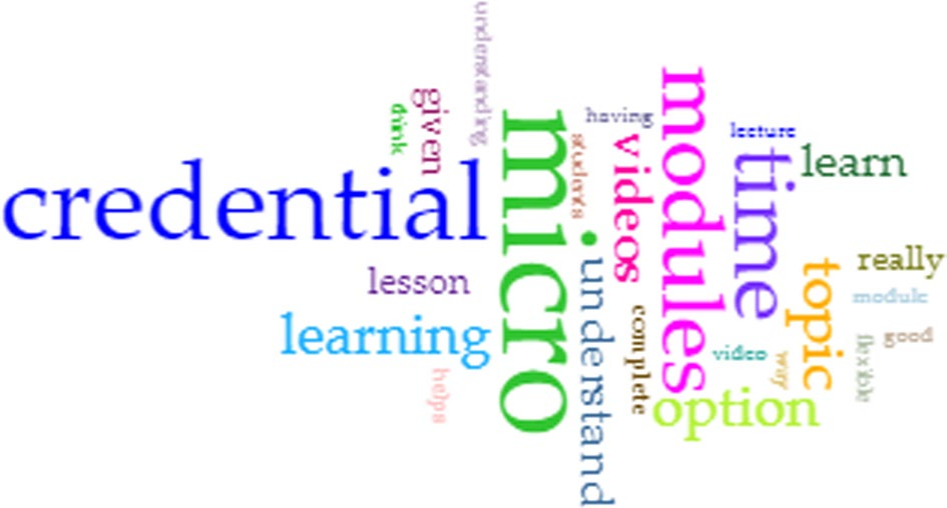
Word cloud for perceived benefits

For the first research question reflecting the perceived benefits, the word cloud from the CirrusTool (Fig. 4) indicated an emphasis on “micro”, “credential” and “modules” which is quite typical when the questions reflect the use of micro-credentials and the modules. The other common words were “time”, “videos”, “learning” and “option”.

**Fig. 5:**
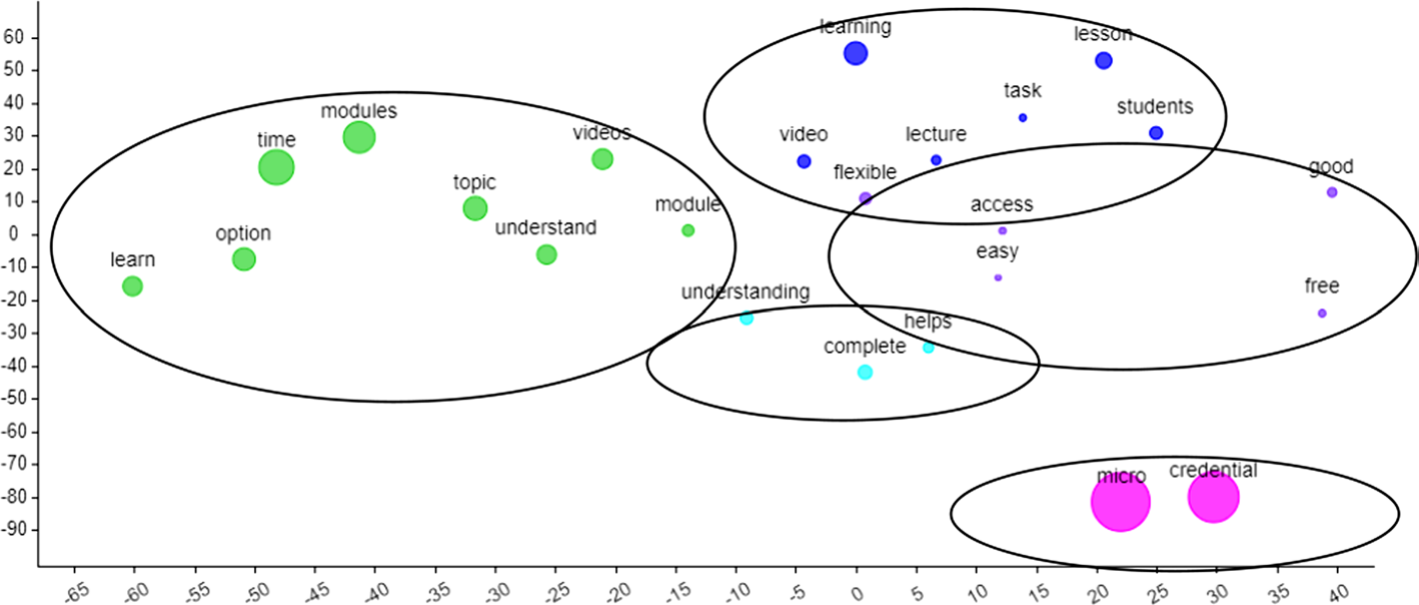
t-SNE generated clusters for the answers to Question 1

Next, referring to Fig. 5, four main clusters beside the “micro” and “credential” cluster. Nevertheless, other clusters reflected that micro-credential provided an option to learning that was flexible and accessible at any time (*green cluster*) in learning the modules or topics mainly due to the availability of various video contents and tasks (*blue cluster*) that help improve their understanding also through the assessment completion (*turquoise cluster*). Furthermore, the flexibility due to ease of use and access is a good option, especially since it is free (*purple cluster*). Example responses are:

*Micro credential makes my learning process easy as the pre-recorded lectures are there, and I can access it anytime I want. Moreover, the exercises given below the lecture videos are very challenging, and it helps me to recall what I learned. Moreover, the certificate provided at the end of the learning motivates me to do more. Overall, micro-credential makes my learning effective.*

*I think it is good because I can use my own time to do all the tasks. I am also able to repeat all the videos as much as I want to understand the topic.*

*In my opinion, micro-credential can be the best online learning platform so far. It is very convenient, easy to use, and it can help student to always be organized. But overall, I really like this platform. It helps students in so many ways. All videos, links, additional resources can be embedded in this single platform, and of course, it is just one click away.*

**Fig. 6:**
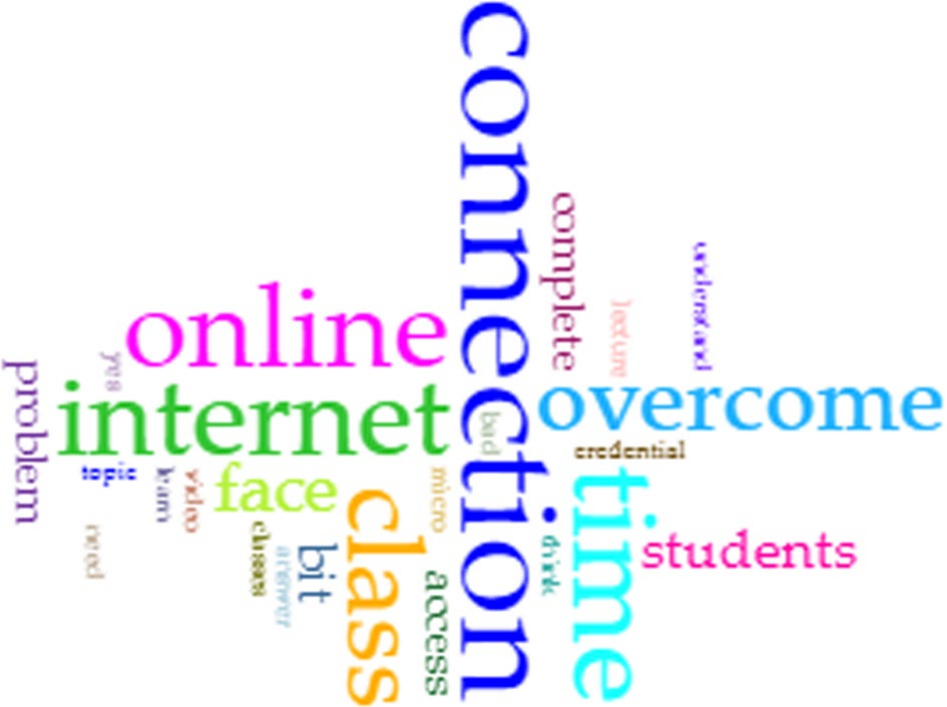
Word cloud for challenges

**Fig. 7:**
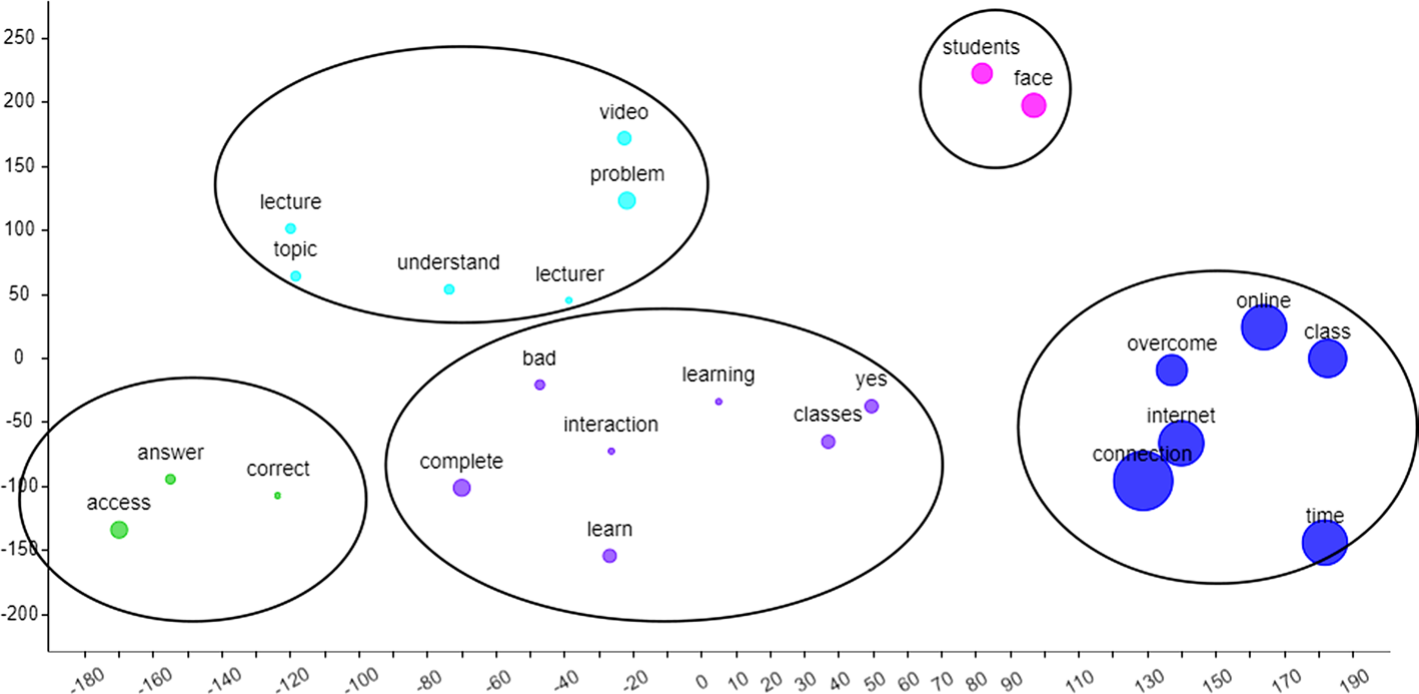
t-SNE generated clusters for the answers to Question 2

As for the question on challenges faced, the word cloud from the CirrusTool (Fig. 6) emphasis “connection”, “online”, “time” and “internet” which indicated connectivity as the main barrier. The scatter plot diagram in Fig. 7 indicated that the main cluster (*blue cluster*) reflected issues with internet connection and accessing the courses online. Next, the respondents also reflected the need to have more face-to-face interactions (*pink cluster*) where they also faced problems in completing the modules (*purple cluster*) due to the system not detecting their completion status.

Furthermore, they indicated issues with the system in playing the uploaded lecturer videos (*turquoise cluster*) and providing assessment feedback (*green cluster*). It was observed that technical issues reflected the main issue, and some respondents stated the following:

*I don’t really feel the real interaction in the class, but I have to learn and adapt this situation so that it will be easier for me to learn.*

*However, it is frustrating that sometimes I had to watch a video multiple times in order for the system to identify that I have done watching the video.*

*What I don’t like is that it is still online learning. I still wish we could have face-to-face classes which is more enjoyable.*

**Fig. 8:**
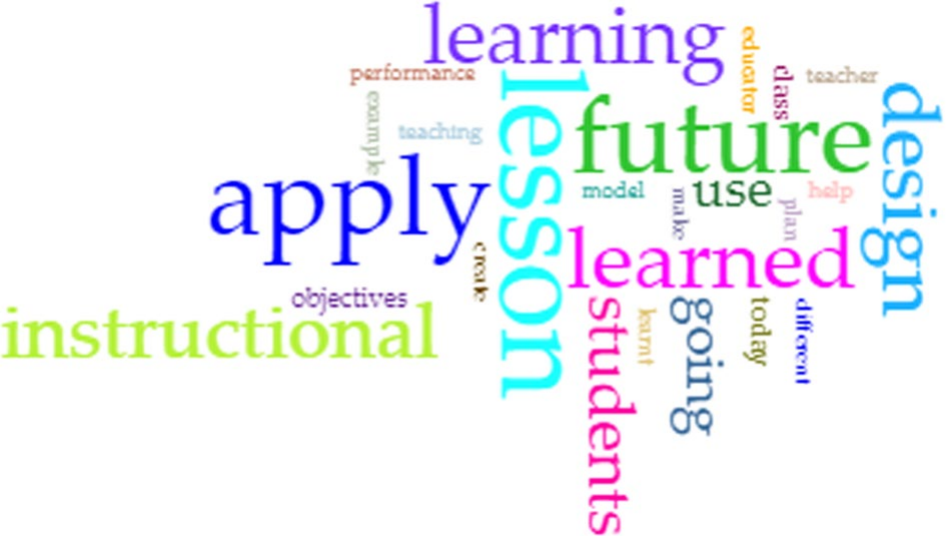
Word cloud for challenges

As for the last question on professional relevance in the future, the word cloud from the CirrusTool (Fig. 8) emphasis “lesson”, “apply”, “future” and “instructional” which indicated they believe the course will be beneficial in developing lessons and instructions in the future.

**Fig. 9:**
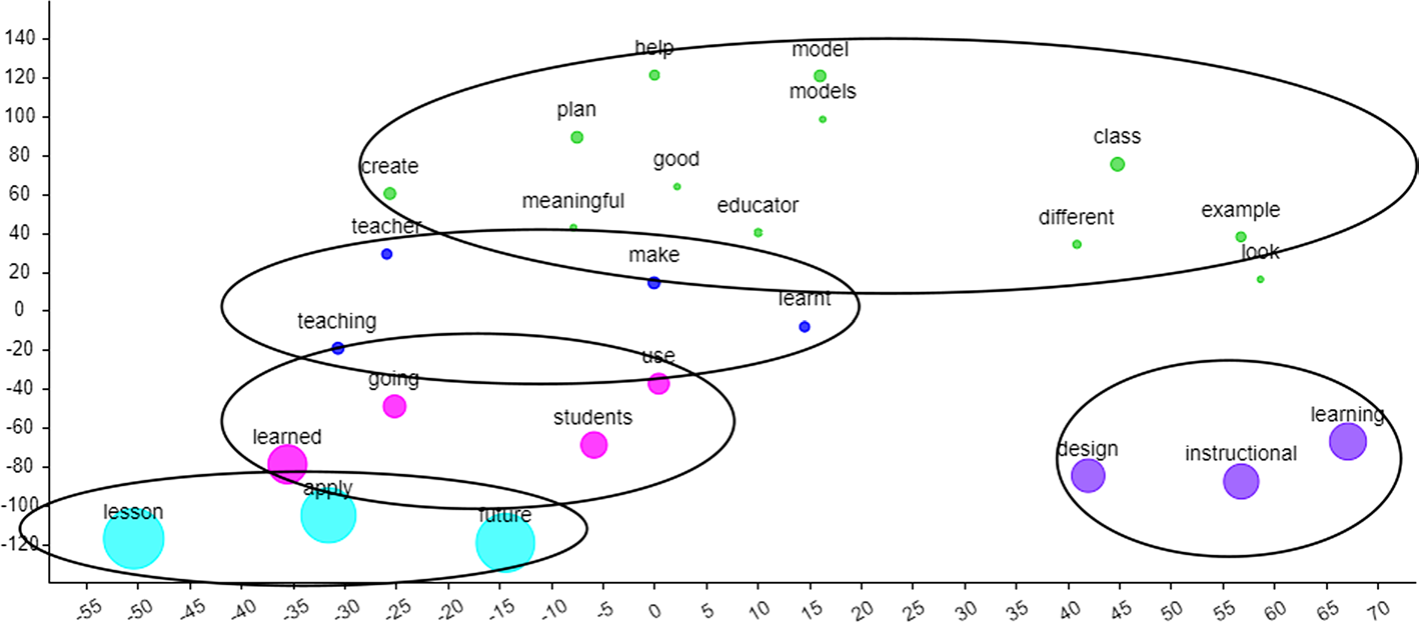
t-SNE generated clusters for the answers to Question 3

In Fig. 9, the main cluster (*turquoise cluster*) reflects that they perceive that they can apply what they have learned in the future, especially how to design instructions for learning (*purple cluster*) and use them for their future students (*pink cluster*). Furthermore, they believe what they have learned will make them better teachers (*blue cluster*), especially by creating a more meaningful learning experience (*green cluster*). Example feedback from the students are:

*I am going to apply everything I’ve learnt in my future lessons once I become a teacher. It is very important to organize the instruction step y step in order to achieve a meaningful lesson*

*I will apply what I have learned in today’s lesson on my future classroom. I would use this to design the lesson.*

*I am going to apply technology skills for future usage in educational sector to ensure the aims to create a higher digital literacy among the students will be achieved. For example, I learned a lot of application/instructional tools that become a great teachers’ aid tools such as flippity.net, ebook creator and micro-credential tasks. Thus, it will help me to apply this knowledge for future class.*

### Quantitative findings

#### Digital learning identity (DLI)

The descriptive statistics for the DLI factors are presented in Table 1 for the 72 respondents. The Cronbach’s alpha (α) value for all factors and the overall DLI were acceptable and indicated that students had a positive attitude towards digital learning (M = 4.329, SD = 0.412) and self-regulated learning (M = 4.251, SD = 0.457). Overall, respondents had a positive digital learning identity (M = 4.157, SD = 0.413) and the results of the bivariate correlations (Table 2) for DLI were found to be highly correlated with Knowledge Source (*r* = 0.851, *p* < 0.001), Knowledge Achievement (*r* = 0.819, *p* < 0.001), and Challenge (*r* = 0.812, *p* < 0.001) in comparison to other factors. These three factors are also highly correlated with each other. Similarly, a high correlation was also seen between attitude, self-regulated learning, and efficacy.

**Table 1:** Descriptive statistics and reliability for DLI factors

DLI factors	Mean	SD	Min	Max	α
1. Attitude	4.329	0.412	3.30	5.00	0.887
2. Self-regulated learning	4.251	0.457	2.40	5.00	0.829
3. Efficacy	4.085	0.551	1.70	5.00	0.929
4. Knowledge Achievement	4.094	0.714	1.00	5.00	0.946
5. Challenge	4.154	0.736	1.00	5.00	0.958
6. Knowledge source	4.029	0.758	1.00	5.00	0.960
Total	4.157	0.413	2.50	5.00	0.956

**Table 2:** Correlations between DLI factors

DLI Factors	Attitude	Self-Regulated Learning	Efficacy	Knowledge Achievement	Challenge	Knowledge Source	DLI
1. Attitude	–						
2. Self-regulated learning	0.655**	–					
3. Efficacy	0.716**	0.748**	–				
4. Knowledge achievement	− 0.008	0.048	− 0.102	–			
5. Challenge	0.015	− 0.024	− 0.094	0.913**	–		
6. Knowledge source	0.020	0.122	− 0.025	0.897**	0.896**	–	
7. DLI	0.455**	0.504**	0.415**	0.819**	0.812**	0.851**	-

**Correlation is significant at the 0.01 level (2-tailed)

#### Constructivist Online Learning Environment Survey (COLLES)

For the COLLES survey, 72 respondents also participated in the study, where it was observed that α value for all factors were acceptable and students had a positive perception towards online learning (M = 4.109, SD = 0.570) (Table 3). While Tutor Support (M = 4.212, SD = 0.643) and Interpretation (M = 4.212, SD = 0.643) were highly perceived, Interactivity (M = 3.965, SD = 0.780) and Peer Support (M = 4.083, SD = 0.760) while were positively viewed, had the lowest mean value. A bivariate correlation (Table 4) performed showed that all factors were highly correlated with the overall outcome, where the highest relationship was found with Interpretation (*r* = 0.858, *p* < 0.001). Interpretation in return was highly correlated with Peer Support (*r* = 0.700, *p* < 0.001) and Tutor Support (*r* = 0.666, *p* < 0.001). Conversely, Tutor Support was highly correlated with reflection (*r* = 0.858, *p* < 0.001) and Peer Support with Interactivity (*r* = 0.671, *p* < 0.001).

**Table 3:** Descriptive statistics and reliability for COLLES factors

COLLES factors	Mean	SD	Min	Max	α
Professional Relevance	4.129	0.640	1.75	5.00	0.816
Interactivity	3.965	0.780	1.25	5.00	0.911
Reflection	4.087	0.720	2.50	5.00	0.919
Tutor support	4.212	0.643	2.75	5.00	0.914
Peer support	4.083	0.760	2.25	5.00	0.905
Interpretation	4.156	0.686	2.00	5.00	0.925
Overall	4.109	0.570	2.74	5.00	0.959

**Table 4:** Correlations between COLLES factors

COLLES factors	Professional Relevance	Interactivity	Reflection	Tutor Support	Peer Support	Interpretation	COLLES
1. Professional Relevance	–						
2. Interactivity	0.402**	–					
3. Reflection	0.571**	0.573**	–				
4. Tutor support	0.585**	0.533**	0.774**	–			
5. Peer support	0.549**	0.671**	0.570**	0.594**	–		
6. Interpretation	0.565**	0.604**	0.655**	0.666**	0.700**	–	
7. COLLES	0.740**	0.782**	0.849**	0.848**	0.820**	0.858**	–

**Correlation is significant at the 0.01 level (2-tailed)

## Discussion and conclusion

Based on the qualitative research questions, respondents confirmed that such a platform provides flexibility and options in learning as online asynchronous learning was a much-needed tool to overcome learning challenges during the pandemic. Conversely, online access and contents such as videos, reading materials, and tasks were also beneficial in improving their learning and understanding of the subject, which was often interrupted due to unstable internet connections for weekly classes conducted through video conferencing platforms. Other issues were associated to technical issues such as progress detection that impacted course completion and inept video control function especially unavailability of the forward and rewind functions. Besides, aggregated results for the assessment or activities were also deemed as important feedback which was absent as the platform was not designed to support such information. In addition, while respondents did perceive the micro-credential platform as easy to use and welcomed the idea of a free tool that is openly accessible, they did indicate that online learning, while necessary, often lacks emotional interaction and connectivity usually avail in a traditional classroom, especially between peers and the instructor. Nevertheless, overall, they perceived that the course and modules did influence their identity as a future teacher as the skills and knowledge gained was deemed beneficial in designing and developing meaningful learning experience.

Next, the DLIS validated that the respondents had a positive attitude (M = 4.329, SD = 0.412) towards digital learning as they understood the importance of such a strategy and were equally motivated to learn through the platform as implied by the self-regulated learning factor (M = 4.251, SD = 0.457). According to Zimmer et al. (2021), self-regulated learning in DLI determines adult learner goal setting, progress monitoring, and reflection regarding their learning process. Henceforth, further validating the challenges faced as an autonomous learner in monitoring learning progress as reflected by the open-ended questions. Besides, the overall DLI score was highly influenced by how technology influences their learning as reflected based on high correlation between factors, namely knowledge achievement, challenges, and knowledge sources. Zimmer et al. (2021) added that knowledge achievement and sources are a fundamental aspect of shaping teaching practices as positive learning experiences with technology often determine motivation and openness towards using such technology in the future. Moreover, we observe that respondents claim that their experiences with micro-credential and what they have learned from the course will aid them in being better teachers in the future especially in creating meaningful learning experiences. Lastly, a significant correlation between learning attitude, self-regulated learning, and efficacy also indicated that they were comfortable and competent in using technology for autonomous learning; hence validating perceptions indicated by the open-ended question related to benefits. However, we stipulate this as an identity developed based on a need to realize learning goals in the current situation which has influence their online learning experience.

Likewise, the usefulness and relevance of the micro-credential were further validated based on the COLLES survey. Firstly, the respondents did portray a positive professional relevance (M = 4.129, SD 0.640), indicating how the course and modules were relevant to their future professional practices, hence validating the third research question. Next, they also indicated high importance in the role of the instructor (tutor support) in terms of stimulating cognition, participation, and communication in an online learning community, as also reported by Aguilar and Brian Duche Pérez (2021). Furthermore, the importance of a learning community reflected by peer and tutor support was deemed vital to micro-credential adaption, especially the role of the instructor in modeling future teachers. Ehlers (2018) describe that the professionalization process as denoted by micro-credentials requires coherence in learning, and while there are works of literature that champion autonomous learning, the role of an advisor or instructor is imperative to the success of the system. This is also reflected in the enriched virtual model where the instructor has a significant role in facilitating remote learning. Henceforth, the outcome of COLLES validated the professional relevance and highlighted the need for instructor guidance or mentoring.

Interestingly, the lack of mention of open badges and certificates also indicated that the respondents were unaware of the benefits (Cirlan & Loukkola, 2020) outside of the course or undervalue the credentials related to participation and completion (Fanfarelli & McDaniel, 2019; Randall & West, 2020) towards their professional development. While it is an important aspect that should be established (Lim & Hassan, 2020), recognized and strategized in Malaysian HEI, teaching credentials are only established through formal academic certification with majoring or minoring in the field of education. Hence, teaching competencies acknowledgment are primarily targeted for continuous professional development (CPD) hours which may not be acknowledged yet, especially without the required collaboration as strategized by the MQF. Nevertheless, as Randall and West (2020) reported, the complication in micro-credentialling the teaching profession revolves around a lack of recognition nor value that governing bodies project for the teaching profession.

Likewise, if we view micro-credentialing and badging based on different credentialing goals, much can be attained based on the value that can be operationalized. According to Fanfarelli and McDaniel (2019), accumulated badges for a completion certification were given ‘value’ through strategizing it as an assignment. While badges may have not always been effective, the ‘idea of badges’ may be compelling, as done in this study to obtain the completion certificate. According to Boud and Jorre de St Jorre (2021) completion can be an important assurance that all learning outcomes have been achieved. Similarly, we agree with Willis, Flintoff, et al. (2016), Willis, Strunk, et al. (2016) that in the education paradigm, badges should motivate students intrinsically to show evidence of learning, and that can be achieved if it is not viewed as a physical badge. Ellis et al. (2016) explained that unvalued badges that are not designed to fit in an existing framework for micro-credentials could routinely fail. Hence, it is crucial to understand the expectations, implementation, and benefits of badges and micro-credentialling to stakeholders (Young et al., 2019).

To conclude, unquestionably, micro-credentials and digital badging have gained much popularity (Ruddy & Ponte, 2019) and can be a valuable addition to HEI (Young et al., 2019), especially during the pandemic. Moreover, we observed a positive attitude and satisfaction towards using micro-credentials in coping with learning challenges authenticated from the pandemic. While the main challenge in using micro-credentials remains to be substandard online connectivity, the internet issue is also why micro-credentials should be in place to complement online learning. Issues in connectivity, especially for weekly virtual classes conducted through video conferencing, have given micro-credentials value. As completion of the courses aided in understanding the contents, it has also shaped their digital learning identity and enable them to reestablish skills required to be a competent educator. Nevertheless, teachers are still challenged by a lack of understanding or need for non-formal credentials outside of the HEI environment. We stipulate this as rooted from Malaysian teachers’ identity as both an educator and a learner. Nonetheless, Boud and Jorre de St Jorre (2021) claimed that micro-credentials have uncovered the unenviable nature of traditional academic programs. However, its success in HEI can only be established if policies and taxonomies are established (Selvaratnam & Sankey, 2021) to create transparency and diversity (Cirlan & Loukkola, 2020) to foster more inclusive access to education (Chakroun & Keevy, 2018). We also gather that the autonomous learning facilitated by the micro-credentials also aided in providing students a safe place to practice and make mistakes which might not be possible in a video-conferencing weekly class, and this may give them confidence in using such approach in the future. Nonetheless, the main issues with HEI students in Malaysia is lack of awareness of such platform especially for personal development (Kumar & Al-Samarraie, 2019) and therefore we stipulate if micro-credentials are incorporated as part of higher education movement, the exposure may be the needed shift towards the success of micro-credentialing even post tertiary education.

### Limitation and future direction

The respondents in this study are pre-service teachers, hence the outcome is limited namely to pre-service teachers’ educational context and may not be analogous to different career paths. Nevertheless, we believe future studies may follow such a strategy as professional qualification and identity is often subjective towards specific industry or career path. Next, due to the novelty in Malaysia, a transferable credit system or framework has not been established at the time of the study, therefore the value of the credentials as described only refers to validation in the context of the course as determined by the instructor. However, as micro-credentials are projected to support online learning in HEI, future studies may explore experimental or predictive design to investigate use behavior, acceptance, and implementation in HEI and professional settings.

Undoubtedly, these initial stages are susceptible to a surge of enthusiasm defined as the novelty effect where its viability in the future is still unknown (Fontichiaro & Elkordy, 2016). However, without a strategically implemented credentialling framework that recognizes and validates skills, it is challenging to measure micro-credentials professional impact. Therefore, a registry for credentials (Gauthier, 2020) is of utmost importance to ensure a successful relationship between HEI and the targeted industries and future studies should be directed towards exploring the impact and effectiveness. Concurrently, while government HEIs are commencing towards a systematic accreditation process that can be widely applied, private education providers and training bodies have taken this opportunity to jumpstart their endeavors independently and often in collaboration with the industry. Hence, creating a competitive market among these providers where professionally recognized institutions or industries such as Google and Microsoft are gaining trajectory by ensuring employment and recognition which is still an aspect that is only address to a certain degree by government based HEI. In view of that, we suggest further studies to explore how collaboration and competition influences perception, use, design, and development of micro-credentials and the viability of such opportunity in bridging the gap between industry and HEI.

Besides, while the idea of nationally or globally recognized stackable credentials is excellent, yet credentialing is, by all means is also a strategy to commercialize education. According to Ralston (2021), the craze of micro-credentialing also forces us to look into the role of open education when the line between HEI and for-profit e-learning industry has become blurred in defining competence and profitability. Hence, we question if HEIs or other institutions will support each other to develop best practices for micro-credentialing to fulfill the global education goal (Sustainable Development Goal 4-SDG4) to provides inclusive and unbiased quality higher education. Therefore, future studies should focus on challenges and opportunities of such collaboration.

## Data Availability

The data that support the findings of this study are available from the corresponding author upon reasonable request.
